# Imaging evaluation of the hip after arthroscopic surgery for femoroacetabular impingement

**DOI:** 10.1007/s00256-017-2665-y

**Published:** 2017-05-02

**Authors:** Julia Crim

**Affiliations:** University of Missouri at Columbia, Columbia, MO USA

**Keywords:** Femoroacetabular impingement, Hip arthroscopy complications, Hip instability, Psoas atrophy

## Abstract

Arthroscopic surgery for femoroacetabular impingement (FAI) is increasingly frequently performed. Initial reports were that complications were very low, but as experience has increased, a number of long-term complications, in addition to factors related to poor clinical outcomes, have been identified. This review describes the normal and abnormal postoperative imaging appearance of the hip after arthroscopy for FAI. Abnormalities discussed include incomplete resection or over-resection of the impingement lesion, heterotopic ossification, cartilage damage, chondrolysis, instability and dislocation, recurrent labral tear, adhesions, psoas atrophy, infection, and avascular necrosis.

## Introduction

Femoroacetabular impingement (FAI) is a clinical syndrome of hip pain and limited motion that many hip specialists believe to be due to morphological abnormalities of the femoral head, acetabulum, or both. First described by Ganz and colleagues, FAI has been identified as a cause of pain, labral tears, juxtalabral cartilage damage, and premature osteoarthritis [1, 2].

Arthroscopy of the hip is increasingly performed for treatment of labral tears and FAI. Among privately insured patients between the ages of 18 and 64, the rate of surgery increased from 3.6 per 100,000 in 2005 to 16.7 per 100,000 in 2013 [3]. There are a number of known short-term and long-term complications of the surgery, including pain due to under-resection of the cam lesion, fracture, heterotopic ossification, cartilage damage, joint instability, adhesions, psoas abnormalities, neuropraxia, and osteoarthritis. This review is designed to familiarize radiologists with the normal and abnormal imaging appearance of the hip following arthroscopic surgery for FAI.

## Imaging evaluation of impingement morphology

Radiographs, CT, and MRI with or without arthrography are all used to detect morphological abnormalities of the proximal femur (cam lesion) or of the acetabulum (pincer lesion) or both (mixed-type impingement). Radiographs include anteroposterior (AP), frog leg lateral, and false profile views. CT scan includes standard axial, sagittal, and coronal reformatted views. Three-dimensional CT images are often useful in characterizing the extent of morphological changes. It cannot be overemphasized that patients may have morphological findings associated with impingement without having the clinical syndrome [4, 5]. Radiologists must be careful in their reports to describe morphological findings only, rather than to imply that the clinical syndrome is present.

**Fig. 1:**
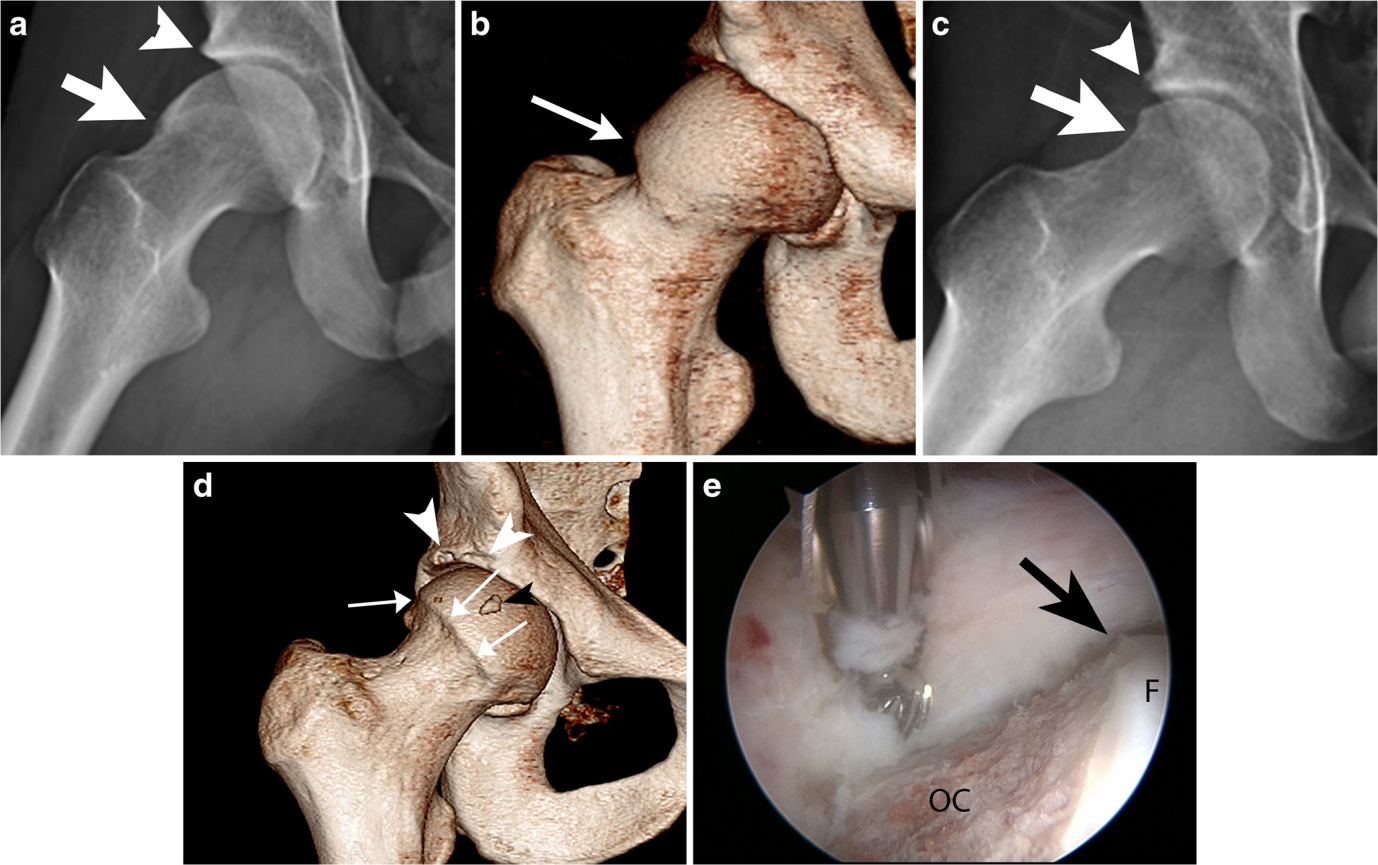
Cam lesion before and after surgery in a 20-year-old man. **a** Preoperative frog leg lateral view shows the anterolateral femoral bony prominence (*arrow*) known as a cam lesion. There is also mild prominence of the anterosuperior acetabular rim (*arrowhead*). **b** Preoperative 3D CT scan shows more fully the extent of the cam lesion at the anterior head–neck junction (*arrow*). **c** Postoperative frog leg lateral view shows osteochondroplasty site (*arrow*). Small focal lucency at the superolateral margin of the acetabulum (*arrowhead*) is the site of the “rim trim” and radiolucent suture anchor placed for labral repair. **d** Postoperative 3D CT scan shows an abrupt contour defect (*arrows*) at the site of surgical resection. This contour change can be mistaken by the unwary for an osteophyte. The “rim trim” and the site of labral reattachment (*white arrowheads*) are also visible. There is a small focus of heterotopic ossification (*black arrowhead*). **e** Arthroscopic image shows osteochondroplasty site (*OC*).* Arrow* points to the margin of osteochondroplasty.* F* femoral head

A cam lesion is a bony prominence anterolaterally or laterally at the junction of the femoral head and neck (Fig. 1). The prominence may be focal, or there may be decreased head–neck offset (pistol grip deformity). When the hip is flexed, adducted, and internally rotated, the bony prominence impinges on the rim of the acetabulum, shearing the labrum superiorly and causing labral tears and paralabral cartilage damage.

**Fig. 2:**
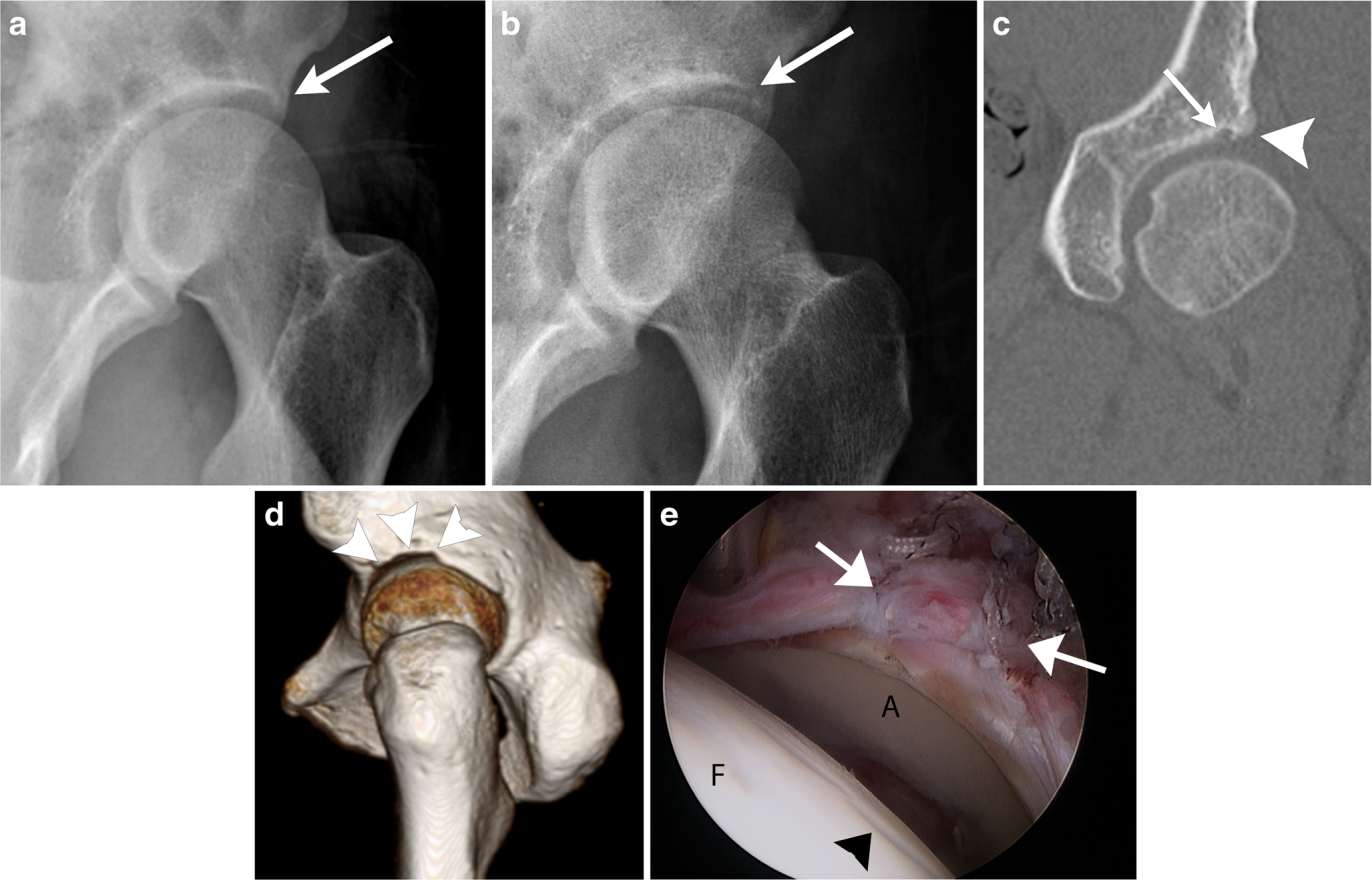
Pincer lesion before and after surgery in a 32-year-old woman. **a** Preoperative false profile view shows prominent contour of the anterior acetabulum (*arrow*). **b** Postoperative false profile view shows subtle bone resection (*arrow*). **c** Coronal postoperative CT shows the bone resection (*arrowhead*) and a lucent track (*arrow*) where a bioabsorbable suture anchor was placed as part of labral repair. **d** 3D postoperative CT best shows the extent of bone resection (*arrowheads*). **e** Arthroscopy picture shows labral repair (*arrows*) after rim trim.* A* articular surface of the acetabulum,* F* femoral head.* Arrowhead* points to articular cartilage damage, probably related to the arthroscopic instruments

**Fig. 3:**
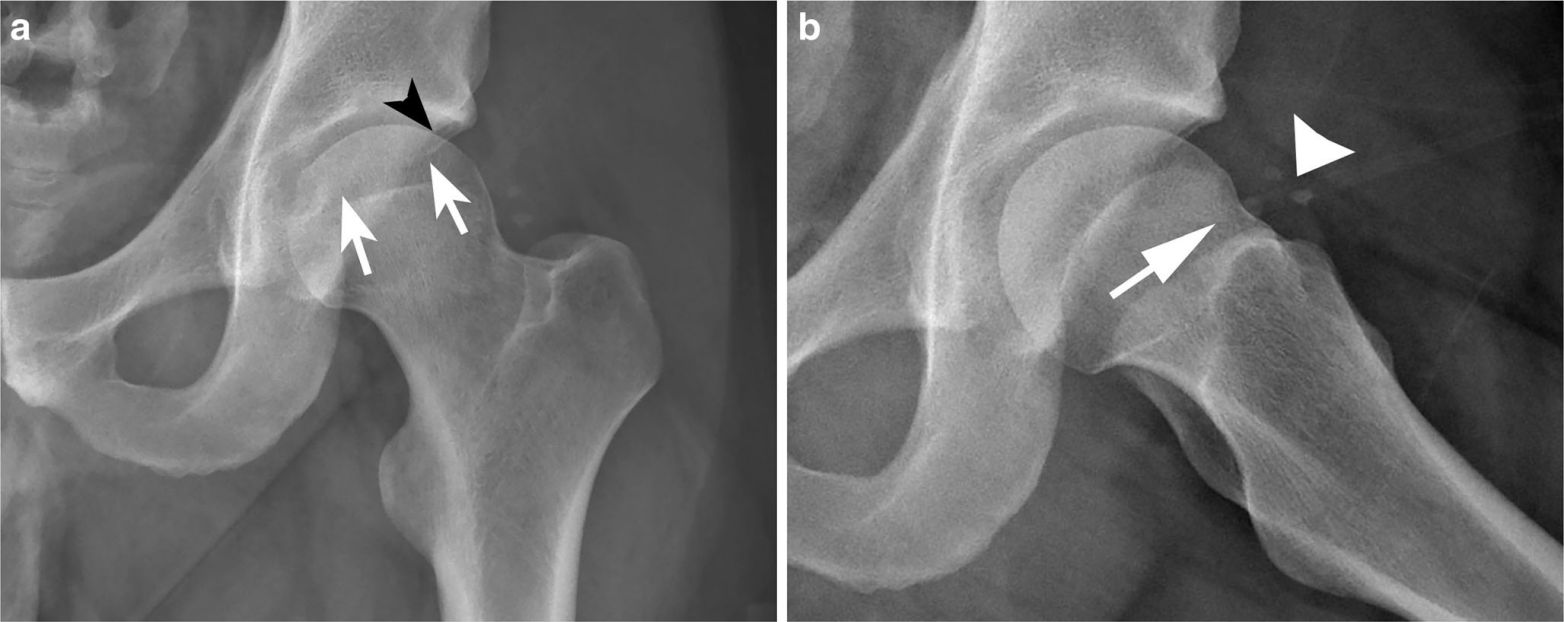
A 22-year-old man with persistent pain after surgery, and imaging suggesting inadequate resection of impingement lesions. **a** Anteroposterior (AP) radiograph shows “crossover sign” indicating persistent retroversion of the superior acetabular rim.* White arrows* show the anterior acetabular rim crossing over the posterior rim (*black arrowhead*). **b** Frog leg lateral radiograph shows a shallow indentation at the femoral head–neck junction representing the surgical resection (*arrow*). The head–neck junction remains prominent. There is also a small amount of heterotopic ossification (*arrowhead*)

A pincer lesion refers to an acetabulum that limits hip flexion and adduction because it extends too far over the femoral head. Femoral head overcoverage may occur owing to an acetabulum that is globally deep (coxa profunda), or there may be a bony prominence at the anterior–superior rim (Fig. 2). On the anteroposterior radiograph, the anterior rim of the acetabulum normally projects medial to the posterior rim. Superior rim retroversion may create a “crossover sign,” where the anterior rim of the acetabulum projects lateral to the posterior rim (Fig. 3a). In either case, the labrum may be pinched between the femoral head and the prominence of the bony acetabulum.

## Arthroscopic technique for FAI

Hip arthroscopy is technically demanding, and a precise surgical technique is key in preventing complications [6]. The hip joint is first distracted by a traction device. Access to the hip is gained via at least two of several standard arthroscopic portals (usually anterior and anterolateral). A vertical capsulotomy may be performed to improve access to the joint, in addition to a horizontal capsulotomy adjacent to the margin of the anterior acetabulum. Once access to the joint is obtained, fluoroscopy is used to locate the cam lesion. The area to be resected may be marked by cautery to allow accurate debridement. A burr is then used to remove the bony prominence. Acetabular labral tears may be repaired, or the labrum may be resected. The surgeon can treat mild pincer impingement by detaching the labrum, trimming the bone, and reattaching the labrum. This procedure is commonly known as a “rim trim.” More severe femoral over-coverage is treated with periacetabular pelvic osteotomy, which lies outside the scope of this article. Chondral lesions may be treated with debridement or a variety of repair techniques [7].

## Normal postoperative imaging appearance of the hip

Postoperative radiographs, computed tomography (CT), and magnetic resonance imaging (MR) after arthroscopic resection of a cam lesion show a sharply angled concave contour (Fig. 1c, d) at the femoral head–neck junction [8, 9]. This angular contour change may be mistaken by the unwary for osteophyte formation. The acetabular “rim trim” may not be visible on radiographs, although a subtle change in contour is sometimes visible. It will be better visualized on CT (Fig. 2) and MRI.

## Outcomes of hip arthroscopy

The literature shows a wide variation in reported outcomes of hip arthroscopy. A 2013 meta-analysis of 92 studies with more than 6,000 patients found that the reported rate of early major complications was 0.58% [10]. The reported major complications included deep infection, skin damage, pulmonary embolus, intra-abdominal fluid extravasation resulting in abdominal compartment syndrome, femoral or femoral circumflex vessel injury, avascular necrosis, femoral neck fracture, dislocation, and death. Minor complications occurred at a rate of 7.5%, and the most common of these were iatrogenic chondrolabral injury and temporary perineal or femoral neuropraxia. A 2016 review of 258 patients found a significantly higher complication rate: 14.34% in the first year after surgery. Major complications occurred in 1.2%, and included femoral neck fracture, septic arthritis, and avascular necrosis of the femoral head [11]. This complication rate is significantly higher than the 4.7% overall knee arthroscopy complication rate reported in over 92,000 cases performed by orthopedic surgeons sitting for part II of the American Board of Orthopedic Surgery [12].

One useful measure of the long-term outcomes of arthroscopic treatment of FAI is the rate at which a second surgery is performed on the affected hip. The second surgery may be revision arthroscopic surgery, open hip surgery for FAI, or conversion to total hip arthroplasty. The data from several studies indicate a significant rate of repeat surgery after arthroscopic surgery for FAI [10, 11, 13–17]. A 2013 meta-analysis found a rate of second surgery after arthroscopic treatment for FAI of 6.3% [11]. The most common second surgery was conversion to total hip arthroplasty. A 2015 meta-analysis found that although functional hip scores improved overall when measured 1 year after index surgery, 14.6% of patients underwent either repeat hip preservation surgery or hip arthroplasty [17]. Most repeat surgeries were performed within 2 years of the index surgery [17]. Repeat surgery often disclosed more than one abnormality. Findings included incomplete resection of the impinging lesion, labral and articular cartilage abnormalities, adhesions, osteoarthritis, and instability [10, 11, 13–17].

Some poor outcomes of hip arthroscopy for FAI are undoubtedly related to patient selection [6]. Patients who have pre-existing osteoarthritis (joint space <2 mm) or are over the age of 50 have worse outcomes of arthroscopy, and a significantly higher rate of conversion to total hip arthroplasty [3, 18–20]. Patients undergoing hip arthroscopy at the ages of 55–64 years have been reported to have a cumulative risk of conversion to total hip arthroplasty of 35% at 5 years [3].

Another outcome measure is patient satisfaction with the results of hip arthroscopy. Only one study could be found that specifically addressed this issue. The authors performed preoperative questionnaires regarding hip function and pain on 86 patients undergoing arthroscopic or mini-open surgery for FAI. They then approached the cohort 12 months postoperatively and asked them to complete new questionnaires [21]. Fourteen percent of the original cohort refused to participate in the 12-month postoperative evaluation because of their degree of unhappiness with the surgery. More than 50% of those who responded stated that they did not have their expectations met for postoperative hip pain, sport, and general physical capacity. It is difficult to know whether the findings of this study reflect technically poor outcomes, poor communication between patients and surgeons, or unrealistic expectations for pain relief.

## Imaging findings of complications of FAI surgery

Many complications of FAI surgery can be seen on imaging studies: heterotopic ossification, incomplete resection or over-resection of the impingement lesion, cartilage damage, rapid osteoarthritis, instability, dislocation, recurrent labral tear, anchor displacement, adhesions, psoas atrophy, infection, and avascular necrosis. Infection and avascular necrosis are not included in this review, as their appearance in this setting is not different than in other settings, and is commonly known. At our institution, small field-of-view images of the affected hip are supplemented with large field-of-view images to evaluate the entire pelvis, to detect causes of hip pain that lie outside the area of surgery.

### Incomplete resection of the impingement lesion

The radiographs and MRI should be scrutinized carefully for residual deformity (Fig. 3). Postoperative radiographs may show a persistent bone prominence, but the patient may still have a successful outcome. As correlation with symptoms is variable, the radiographic report should be descriptive rather than implying the cause of symptoms.

### Over-resection of the impingement lesion

If the surgeon is overly aggressive in removing a cam lesion, the femoral neck may be at an increased risk for fracture. However, there are no data on how much resection increases fracture risk. Risk of stress fracture of the femoral neck has been estimated at 0.07%, with an increased risk in patients who did not follow postoperative weight-bearing restrictions, and in women over the age of 50 [22]. If the pincer lesion is overly resected, the patient may develop anterior instability [23].

### Heterotopic ossification

**Fig. 4:**
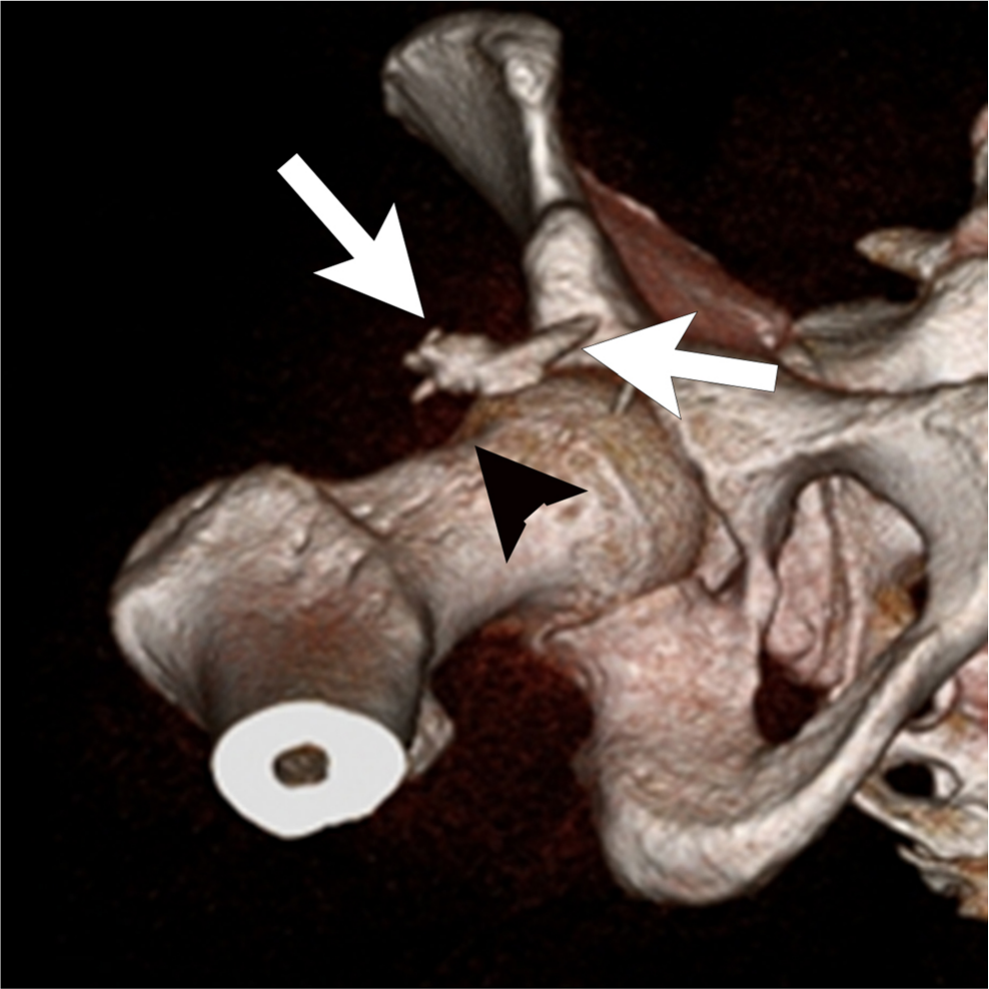
A 25-year-old man with a mild limitation of motion after arthroscopic resection of a cam lesion. 3D CT shows a sheet of heterotopic ossification (*arrows*) anterior to the femoral osteochondroplasty site (*arrowhead*)

Hip arthroscopy carries a much higher risk of heterotopic ossification than arthroscopy of other joints, and is reported in 5–40% of patients, with a decreased rate after administration of indomethacin. [24, 25]. Only about 1% of cases of heterotopic ossification require surgical resection. Heterotopic ossification may be asymptomatic, but may cause pain, impingement, and decreased range of motion. Radiographs and CT are used to make the diagnosis and define the extent of the ossification (Figs. 3, 4).

### Cartilage damage

**Fig. 5:**
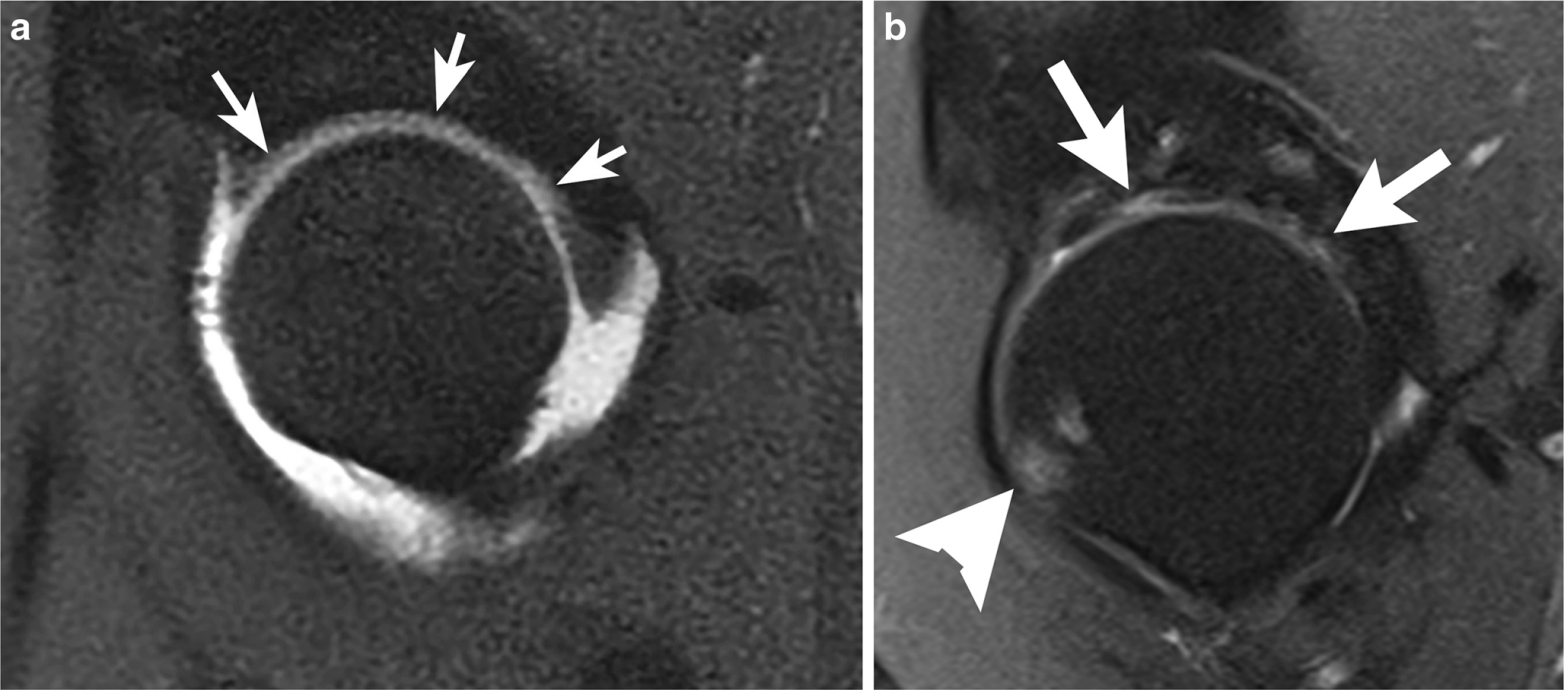
A 30-year-old woman with pain 1 year after arthroscopy. **a** Preoperative sagittal proton density-weighted fat-saturated (PDFS) MR arthrogram at 1.5 T shows homogeneous, intermediate gray articular cartilage (*arrows*) with a small amount of quantum mottle. **b** Postoperative sagittal PDFS MRI at 1.5 T shows fluid extending into acetabular cartilage defects (*arrows*), including a small region of cartilage delamination. It is impossible to know on MRI whether this represents progression of disease or iatrogenic damage occurring at the time of arthroscopy.* Arrowhead* points to bone marrow edema in the femoral head, suggesting ongoing damage in this region as well. Edema due to surgery would be expected to resolve in 4–6 months

Arthroscopic instruments may cause focal damage to joint cartilage (Figs. 2e, 5). Cartilage changes seen on MRI may also reflect cartilage repair techniques. Correlation with the surgical report is important in reaching the correct diagnosis.

**Fig. 6:**
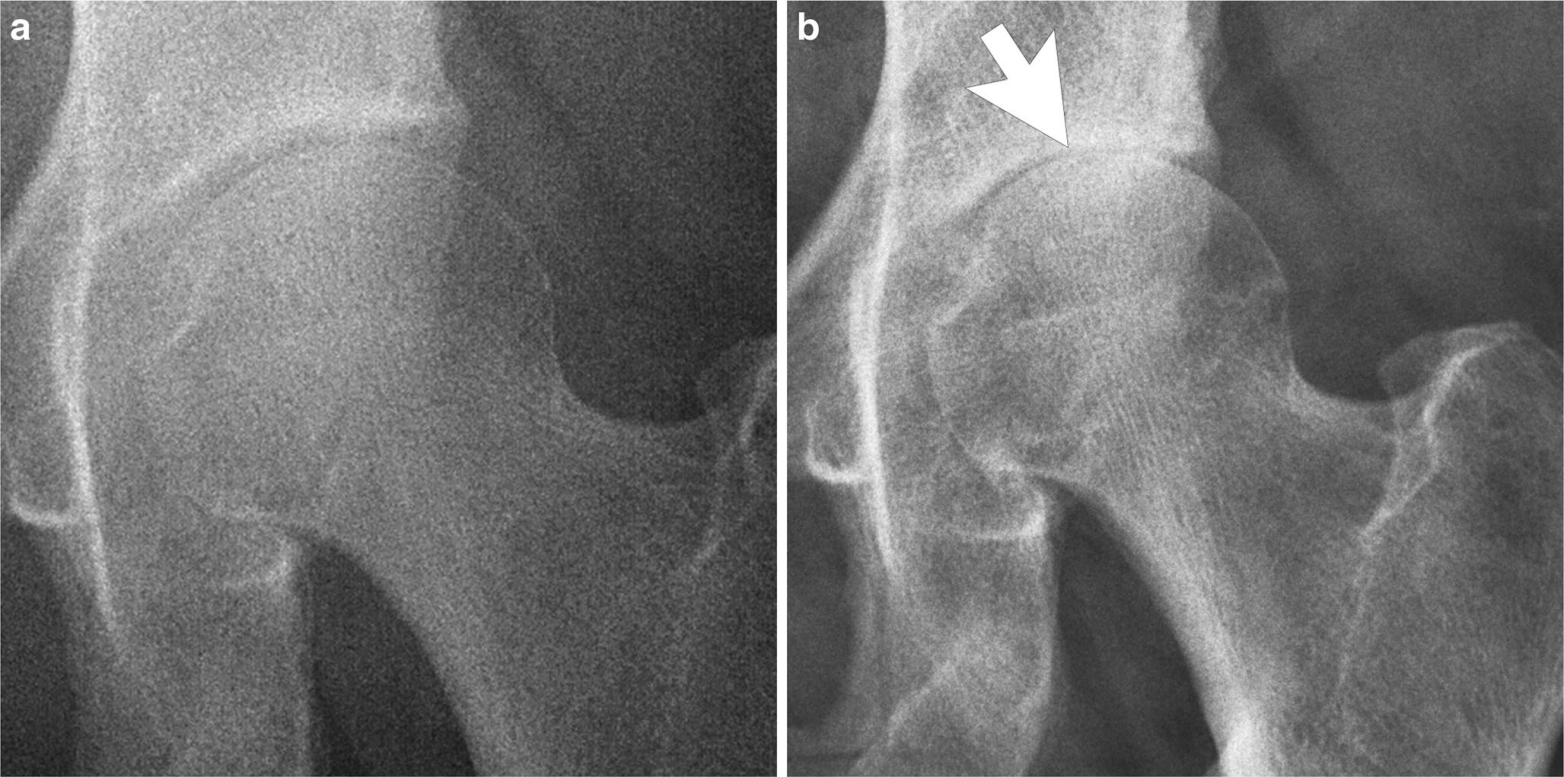
Postsurgical chondrolysis. A 51-year-old woman with a labral tear. Preoperative radiographs were performed elsewhere, and were reportedly normal. She had severe, progressive pain following surgery that was not relieved by narcotic medication. Joint aspiration was negative for infection. **a** AP weight-bearing radiograph 4 months postoperatively shows mild joint space narrowing. **b** AP weight-bearing radiograph 8 months after arthroscopy shows progressive, uniform joint space narrowing (*arrow*), but no osteophytes. This radiographic finding is indicative of chondrolysis or infection or inflammatory arthritis

Occasionally, chondrolysis may occur. Chondrolysis refers to diffuse cartilage resorption due to mechanical, thermal or chemical causes. It has been reported to occur after arthroscopy [26]. On radiographs, it is characterized by rapid, diffuse joint space narrowing and the absence of osteophytes (Fig. 6). Infection must always be excluded, and if chondrolysis is suspected then joint aspiration is recommended.

### Labral abnormalities

**Fig. 7:**
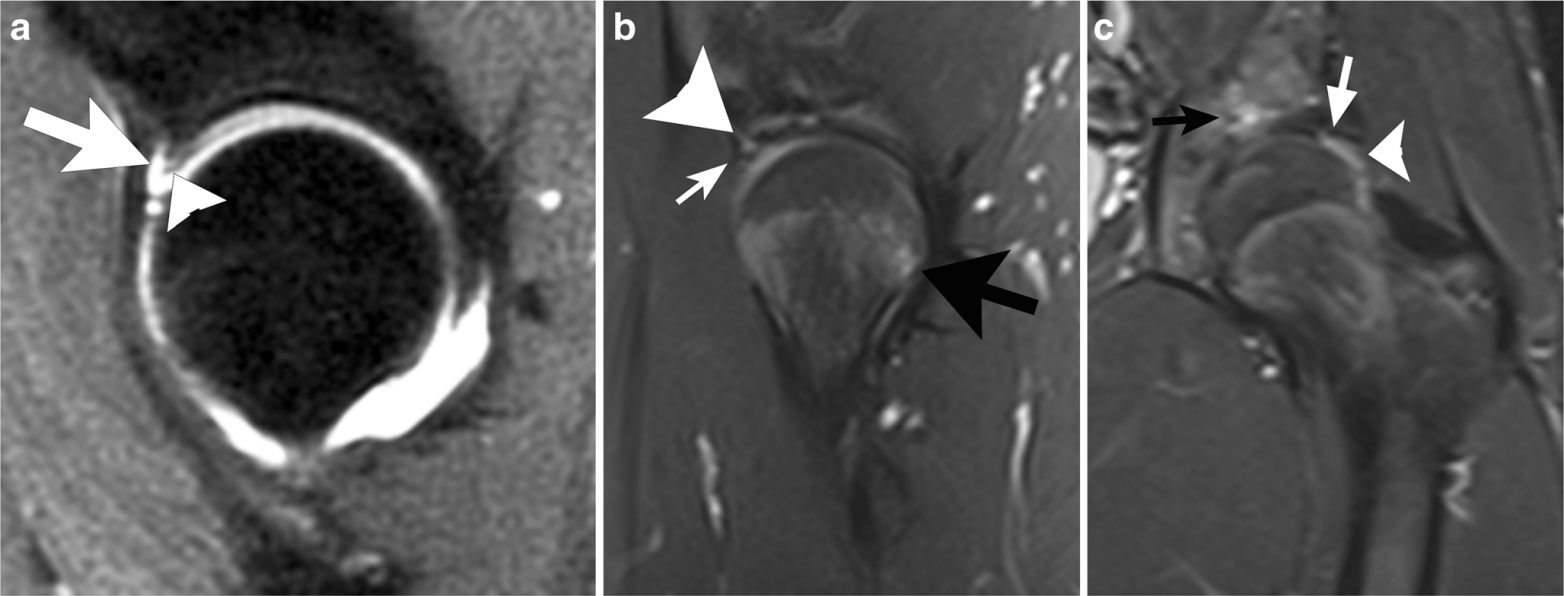
An 18-year-old man underwent femoroacetabular impingement (FAI) surgery including repair of a labral tear, and presented 1 year later with recurrent pain. **a** Preoperative sagittal PDFS MR arthrogram at 1.5 T shows a labral tear (*arrow*). **b** Sagittal PDFS MR arthrogram at 1.5 T obtained 1 year postoperatively shows fluid (*arrowhead*) between the diminutive labrum (*white arrow*) and the bone. The acetabular contour is altered because of rim trim performed at the time of labral repair. Bone marrow edema is present in the femoral neck (*black arrow*) consistent with stress response. **c** Coronal STIR MR arthrogram at 1.5 T 1 year postoperatively shows a loss of articular cartilage (*white arrow*) and an absent labrum (*arrowhead*) at the site of prior repair, in addition to acetabular bone marrow edema (*black arrow*)

**Fig. 8:**
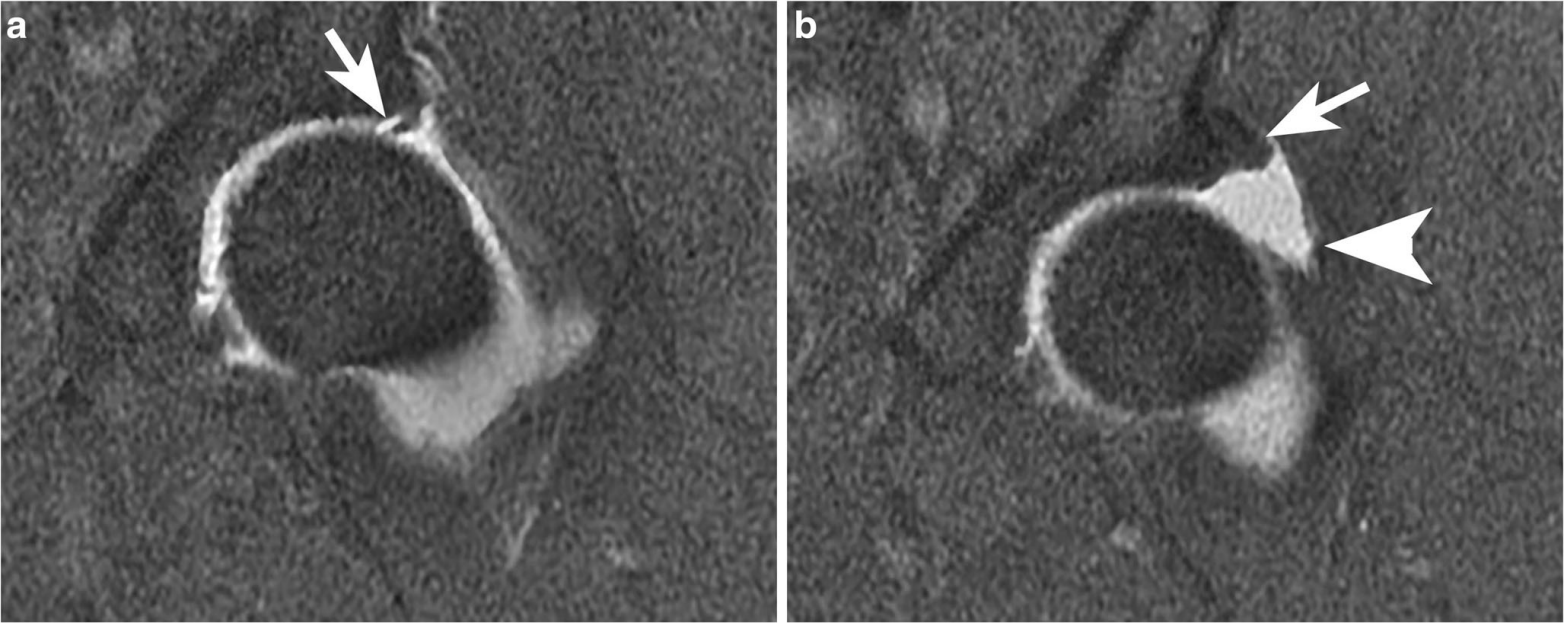
A 35-year-old woman with labral displacement after surgery. **a** Preoperative coronal T1-weighted fat-saturated T1FS MR arthrogram at 1.5 T shows a tear of the anterosuperior labrum (*arrow*). **b** Postoperative coronal T1FS MR arthrogram at 1.5 T shows that the labrum has displaced superiorly (*arrow*) and is adherent to the lateral margin of the ilium immediately above the articular surface. Capsule defect (*arrowhead*) is also evident

A torn labrum is usually repaired at the time of arthroscopy for FAI. A failed labral repair is commonly found at 2nd look arthroscopy, but may be difficult to distinguish on MRI from operative changes. On postoperative MRI, the labrum often appears small, with an irregular contour [27]. One study, which was performed without surgical correlation, found that abnormalities thought to represent recurrent tear at MR arthrography may be asymptomatic [28]. It should be remembered that labral tears in the general population may also be asymptomatic [29]. In the author’s experience, the best clues to recurrent tear, just as in recurrent tears of the meniscus of the knee, are separation of fragments, tear in a new location, and a paralabral cyst. Comparison with preoperative MRI is very useful (Figs. 7, 8).

Obliteration of the paralabral sulcus is very common in asymptomatic patients [28] and should be considered a normal postoperative finding.

**Fig. 9:**
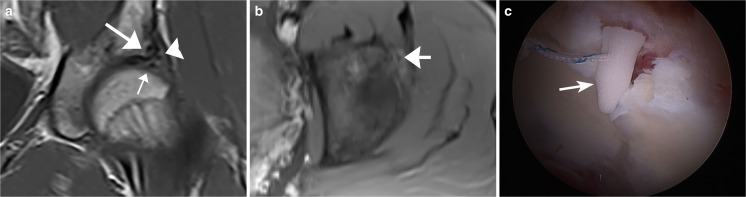
A 17-year-old boy with a catching and locking sensation 3 months after labral reattachment. **a** Coronal T1WI at 1.5 T shows a detached labrum (*small arrow*), the site of suture anchor placement (*large arrow*), and a nonspecific dark structure (*arrowhead*) that could represent a scar, but correlated with a displaced suture anchor at the time of arthroscopy. **b** Axial PD-weighted image at the same time as** a** shows the displaced anchor (*arrow*). The MRI findings were prospectively interpreted as possible displaced surgical suture material adjacent to the labral repair. **c** Arthroscopic image shortly after MRI shows that the suture anchor (*arrow*) has displaced from the acetabulum

Repair of a labral tear is usually performed with suture anchors and sutures. A displaced suture anchor can cause pain, and may be detected on MRI as a small focus of low-signal intensity (Fig. 9).

Absence of the labrum on MRI in a postoperative hip may indicate attrition of a repaired labrum, or a resected labrum. As labral resection is an accepted surgical option, it is prudent for the radiologist to review the surgical report in a postoperative patient. A small study with a 10-year follow-up found that patients with labral resection vs labral repair had no difference in pain with impingement maneuver [30]. In this study, revision arthroscopic surgery was performed in 24% of patients who had been treated with labral repairs, but in 54% of patients who had undergone labral resection. Rates of conversion to THA were equal in both groups.

### Instability

The hip joint owes much of its anterior stability to the iliofemoral ligament (ligament of Bigelow). The iliofemoral ligament protects the hip against hyperextension, external rotation, and anterior displacement [31]. It arises from the inferior margin of the anterior inferior iliac spine, immediately inferior to the straight head of the rectus femoris, and has two limbs that diverge in an inverted V shape to insert on the anterior intertrochanteric ridge, one medially and one laterally. Arthroscopic portals and capsulotomy may compromise the integrity of the iliofemoral ligament [32, 33]. Many arthroscopists close the capsule at the conclusion of the surgery to improve joint stability [34]. With or without capsule closure, the iliofemoral ligament may be compromised, leading to anterior subluxation and sometimes to dislocation.

Signs and symptoms of anterior hip instability are nonspecific. Patients complain of anterior pain, and sometimes of a “popping” or a sensation of “giving way”. They may show apprehension when the hip is extended.

**Fig. 10:**
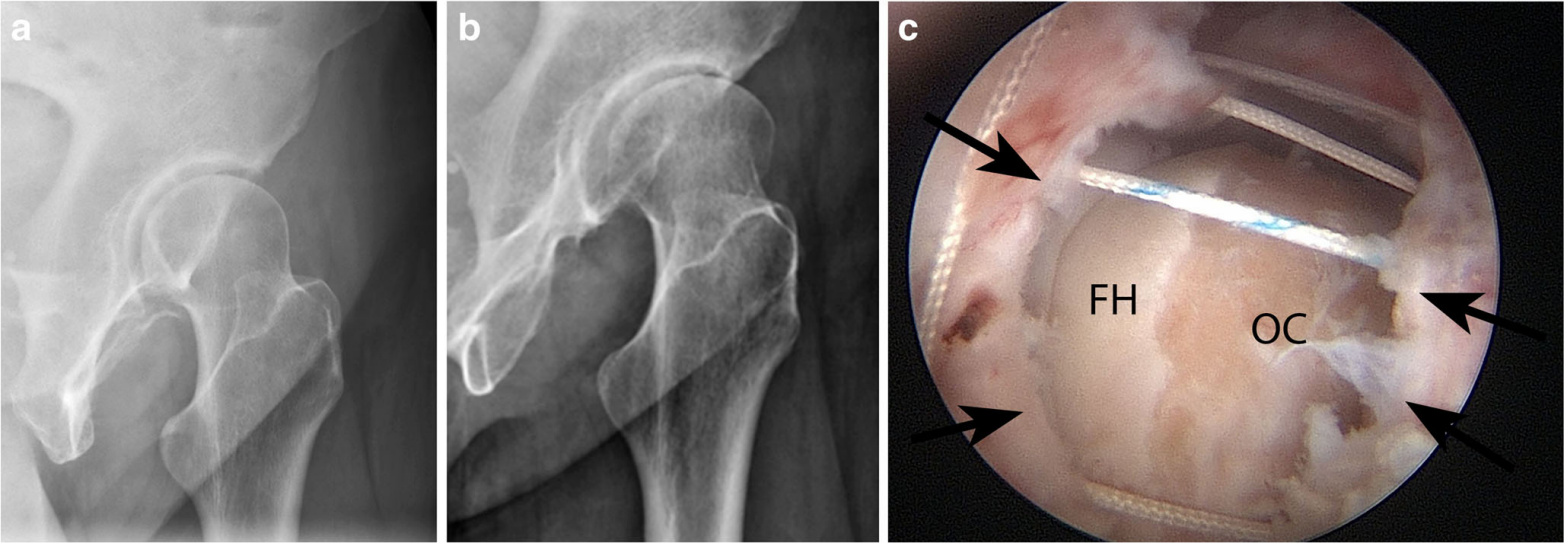
A 23-year-old woman with a sensation of “giving way” after arthroscopy. The standing false profile view, obtained with the pelvis rotated 45° posterior oblique, is best to demonstrate anterior instability. The patient was treated with capsule closure. **a** Normal preoperative false profile view shows the femoral head situated concentrically within the acetabulum. **b** Postoperative false profile view shows increased posterior joint space, and anterior subluxation of the femoral head. **c** Arthroscopy image shows sutures placed for capsule closure.* FH* femoral head,* OC* osteochondroplasty site,* black arrows* point to separated margins of the anterior joint capsule

Anteroposterior radiographs are usually normal except when frank dislocation occurs. The standing false profile view may show widening of the posterior joint space and uncovering of the anterior femoral head (Fig. 10).

Magnetic resonance imaging or MR arthrography shows a defect in the iliofemoral ligament (Fig. 10) that permits anterior subluxation. Some patients may have asymptomatic capsule defects [28].

Another possibility to consider is that unrecognized hip instability may have been present before hip arthroscopy. Hip instability in the absence of previous surgery was long considered to be rare, but is increasingly recognized [32, 35]. FAI has also been suggested as a cause of posterior hip instability [36, 37].

### Hip dislocation

**Fig. 11:**
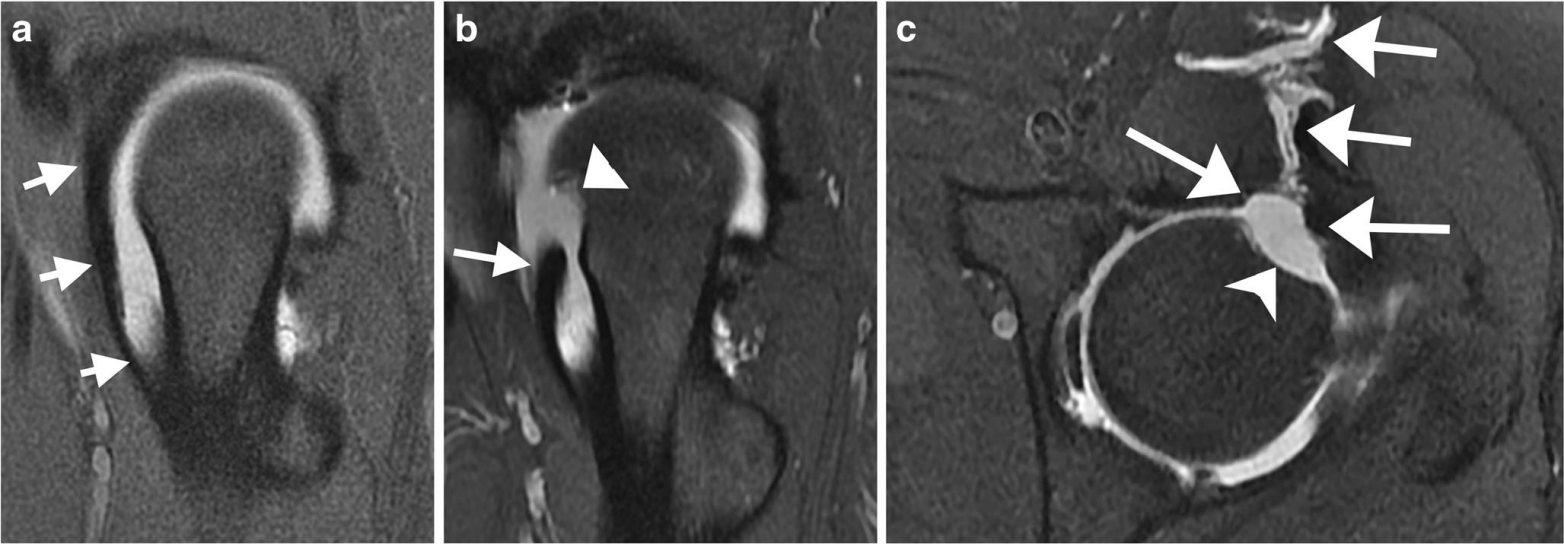
A 27-year-old woman complaining of ongoing pain and subjective instability after arthroscopy for FAI. **a** Preoperative sagittal PDFS MR arthrogram at 1.5 T shows intact iliofemoral ligament (*arrows*). **b** Postoperative sagittal PDFS MR arthrogram at 1.5 T at the same location as** a** shows the discontinuity of the iliofemoral ligament (*arrow*). The osteochondroplasty defect (*arrowhead*) appears unremarkable. **c** Postoperative axial PDFS MR arthrogram at 1.5 T shows the capsule defect (*arrows*) with contrast medium extending through the defect. Careful attention to the injection technique is needed to avoid extra-articular placement of contrast mimicking extravasation.* Arrowhead* points to osteochondroplasty site

**Fig. 12:**
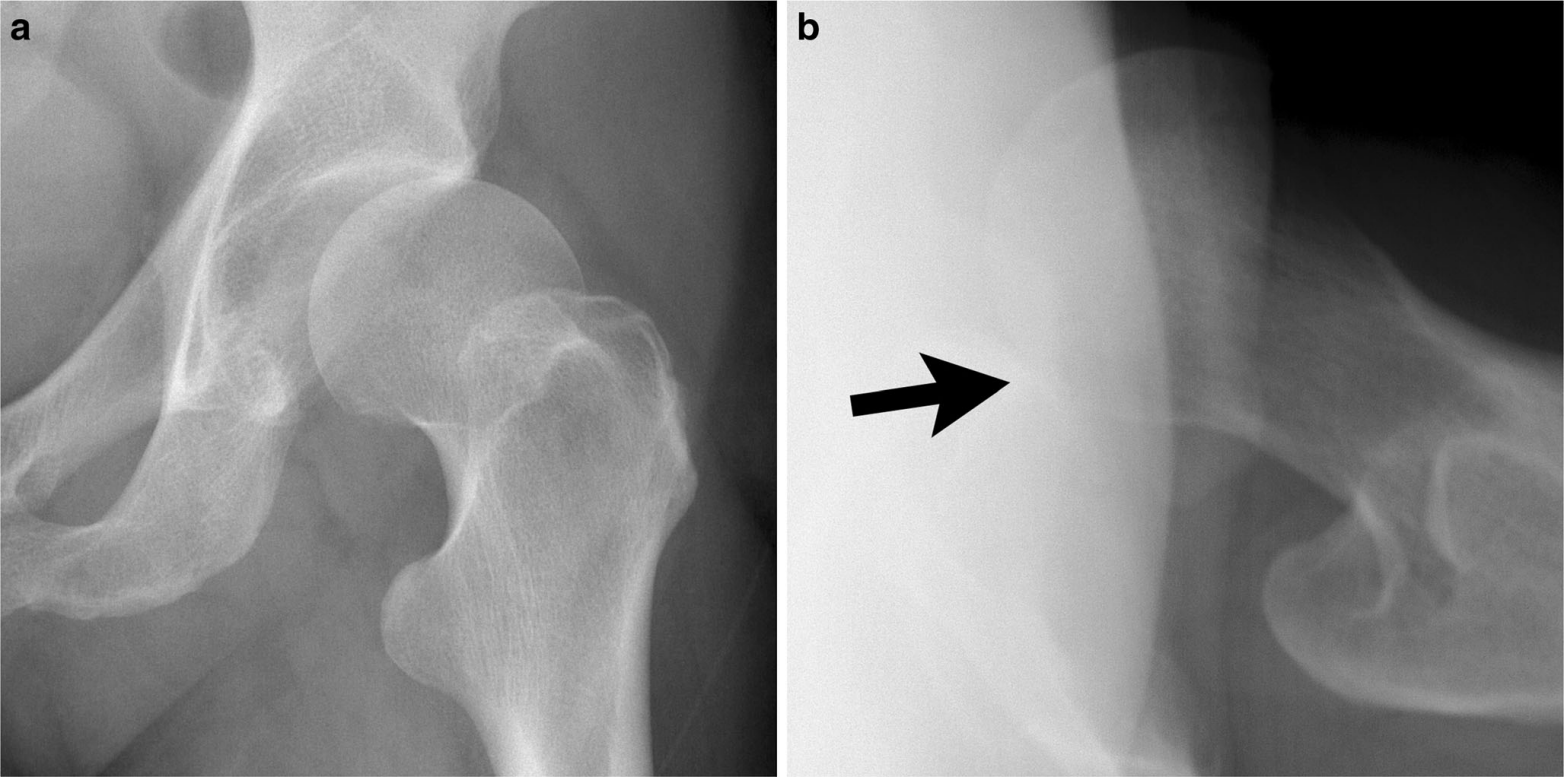
A 50-year-old man had an atraumatic anterior hip dislocation following arthroscopy for FAI. He subsequently underwent total hip arthroplasty. **a** AP radiograph shows the femoral head perched on the rim of the acetabulum. **b** Cross table lateral radiograph confirms that the dislocation is anterior. The femoral head is perched on an anterior acetabular rim (*arrow*)

Most traumatic hip dislocations are posterior hip dislocations due to a posterior force on a flexed hip (“dashboard injury”). In contrast, hip dislocations after arthroscopy usually occur in the anterior direction. They are often due to minor trauma (Fig. 11) and are most likely related to iatrogenic instability. They have occurred despite surgical closure of the joint capsule, and have been associated with acetabular undercoverage (native or due to surgical resection), labral debridement, capsular insufficiency, or iliopsoas tenotomy (Fig. 12 [23]).

### Progressive osteoarthritis

Osteoarthritis may be seen to progress rapidly after hip arthroscopy. This may have multiple causes. Direct trauma by arthroscopic instruments to the cartilage is one cause. Another cause of rapidly-developing osteoarthritis is iatrogenic instability, and a third is progression of previous osteoarthritis.

### Psoas abnormalities

Arthroscopic release of the psoas tendon is a common procedure performed during arthroscopy to alleviate iliopsoas impingement [38]. The psoas tendon is readily visible on MRI at the level of the hip as a black, round structure lying posterior to the iliacus muscle. A recent study found that compared with patients who did not undergo release, patients who had undergone release had significant psoas atrophy on MRI evaluation, which corresponded to measurable muscle weakness [39].

**Fig. 13:**
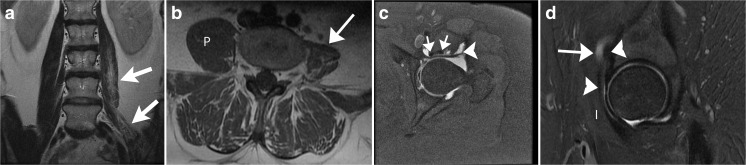
A 40-year-old woman underwent lumbar spine MRI for evaluation of left lower extremity weakness. The psoas findings were unexpected and prompted a medical records search, confirming previous psoas release at the time of hip arthroscopy, and increasing weakness dating from that surgery. There were no spinal abnormalities to cause the psoas atrophy. Hip MR arthrogram was subsequently performed. **a** Coronal T2 MRI at 1.5 T shows marked atrophy of the left psoas muscle (*arrows*) relative to the right. The muscle is small, and streaks of fat are interspersed with the muscle fibers. **b** Axial T1-weighted MRI at 1.5 T shows that the left psoas (*arrow*) is smaller than the right, and contains high signal intensity fatty streaks consistent with atrophy.* P* normal right psoas muscle. **c** Axial T1FS MR arthrogram at 1.5 T shows the surgically split psoas tendon (*arrows*). Also note the anterior joint capsule defect (*arrowhead*); this patient did not have instability symptoms. **d** Sagittal PDFS MR arthrogram at 1.5 T shows fluid (*arrow*) tracking along the psoas tendon (*arrowheads*). The iliacus (*I*), anterior to the psoas tendon, appears normal

Psoas atrophy after arthroscopic release may be profound, and may involve the iliacus muscle as well (Fig. 13). This finding may be seen on MRI of the lumbar spine, in which case the history given to the radiologist often will not mention the history of hip arthroscopy. Because the psoas is innervated by multiple spinal levels, the finding of atrophy involving the entire psoas should raise consideration of psoas release as a more likely etiology for the atrophy than an abnormality in the lumbar spine.

Psoas weakness may occur, even when an arthroscopic release is not performed. A study of 8 patients who underwent arthroscopy without psoas release found that they had persistent psoas weakness 2.5 years after surgery [40]. The strength of other muscle groups was normal.

### Adhesions

**Fig. 14:**
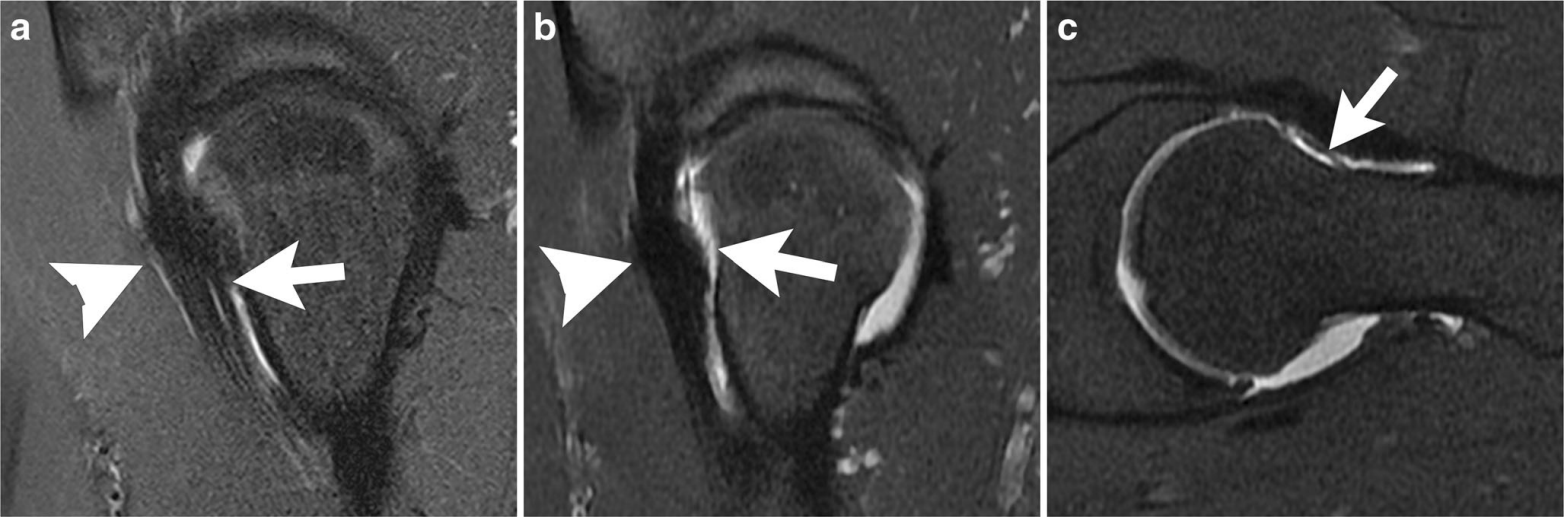
A 30-year-old man with a sensation of tightness and pain when fully extending his hip. The pain had been gradually increasing in the 1.5 years since his arthroscopy for FAI. **a** Sagittal PDFS MR at 3 T shows that the iliofemoral ligament is thickened (*arrowhead*) and there appears to be a fibrous strand (*arrow*) extending to the osteochondroplasty site. The surgeon felt that this might be a spurious finding related to the lack of joint distention; thus, MRI arthrography was subsequently performed. **b** Sagittal PDFS MR arthrogram at 1.5 T. Contrast material slightly separates the thickened iliofemoral ligament (*arrowhead*) from the anterior margin of the femoral neck, and facilitates recognition of fine strands between the capsule and the femoral head. **c** Axial oblique T1FS MR arthrogram at 1.5 T best shows the fibrous adhesions (*arrow*)

Adhesions may form between the joint capsule and the osteochondroplasty site, and present with groin pain and a sensation of “tightness.” They are visible on MRI or MR arthrography (Fig. 14) as irregular bands of fibrous tissue, usually adjacent to the osteochondroplasty site [41].

### Infection and avascular necrosis

These complications are fortunately rare, but should always be on the radiologist’s checklist in evaluating any musculoskeletal imaging study.

## Failure due to incorrect initial diagnosis

If a patient has persistent pain after surgery for FAI, the possibility that the initial pain was due to a different cause than FAI should be considered. Cam and pincer type morphology are very common and may be asymptomatic [4, 5]. Pain due to osteoarthritis, athletic pubalgia, muscle/tendon tears, ischiofemoral impingement, or iliopsoas impingement may be erroneously ascribed to FAI. A new problem may also emerge following successful arthroscopy. The radiologist interpreting a preoperative or postoperative study should be alert for other abnormalities which may cause hip pain.

## Conclusions

The radiologist plays an important role in the evaluation of the hip after arthroscopy. A checklist of possible abnormalities is useful to ensure complete evaluation and accurate diagnosis.
